# Modeling the Effects of Temperature and Resource Quality on the Outcome of Competition Between *Aedes aegypti* and *Aedes albopictus* and the Resulting Risk of Vector-Borne Disease

**DOI:** 10.1007/s11538-025-01518-x

**Published:** 2025-08-30

**Authors:** Emma Beck, Lauren Beuerle, Patt Martin, Regan Stambaugh, Rebeca de Jesús Crespo, Michael A. Robert, Suzanne L. Robertson

**Affiliations:** 1 Department of Mathematics, University of Washington, Seattle, WA USA; 2 Department of Mathematics and Statistics, Elon University, Elon, NC USA; 3 Department of Mathematics, University of North Carolina Asheville, Asheville, NC USA; 4 Department of Mathematics and Statistics, Grinnell College, Grinnell, IA USA; 5 Department of Environmental Sciences, Louisiana State University, Baton Rouge, LA USA; 6 Department of Mathematics, Virginia Tech, Blacksburg, VA USA; 7 Center for the Mathematics of Biosystems (VT-CMB), Virginia Tech, Blacksburg, VA USA; 8 Center for Emerging Zoonotic and Arthropod-borne Pathogens (CeZAP), Virginia Tech, Blacksburg, VA USA; 9 Department of Mathematics and Applied Mathematics, Virginia Commonwealth University, Richmond, VA USA

**Keywords:** Stage-structure, Competition, Dengue virus, Temperature, Differential equations

## Abstract

The community composition of vectors and hosts plays a critical role in determining risk of vector-borne disease transmission. *Aedes aegypti* and *Aedes albopictus*, two mosquito species that both transmit the viruses that cause dengue, chikungunya, and Zika, share habitat requirements and compete for resources at the larval stage. *Ae. albopictus* is generally considered a better competitor under many conditions, while *Ae. aegypti* is able to tolerate higher temperatures and is generally a more competent vector for many pathogens. We develop a stage-structured ordinary differential equation model that incorporates competition between the juvenile stages of two mosquito populations. We incorporate experimental constraints on competition coefficients for high and low quality food resources and explore differences in the potential outcomes of competition. We then incorporate temperature-dependent fecundity rates, juvenile development rates, and adult mortality rates for each species, and we explore competition outcomes as a function of temperature. We show that regions of coexistence and competitive exclusion depend on food quality and relative values of temperature-dependent life history parameters. Finally, we investigate the combined impacts of temperature and competition on the potential for dengue transmission, and we discuss our results in the context of present and future risk of mosquito-borne disease transmission.

## Introduction

Environmental factors have been extensively used as indicators of mosquito-borne disease risk mapping at large spatial scales (Kitron 2000; Blagrove et al. 2017; Sallam et al. 2017). Our understanding of vector ecology and the thermal preferences of different mosquito vector species has been important for understanding current trends and predicting future risks associated with climate change. Research on mosquito population range shifts suggests that increases in global surface temperatures will lead to wider distributions of important disease vectors (Hales et al. 2002; Iwamura et al. 2020). While current ranges and future projections are helpful for disease prevention strategies, they provide a coarse view of risks based on broad assumptions, including the potential presence of mosquitoes in a given environment (Kitron 2000). For cities that fall within the climatic range of medically important mosquitoes, microclimate variation and resource availability at finer scales are two factors that may drive heterogeneity in neighborhood distribution (Evans et al. 2019; Wimberly et al. 2020), a scale relevant for vector control in practice.

Two medically important species that share habitat requirements broadly are *Aedes aegypti* (Linnaeus) and *Aedes albopictus* (Skuse). While *Ae. aegypti* has been in the Americas since European settlement (Powell and Tabachnick 2013), *Ae. albopictus* was first detected in the United States in 1985 and has quickly expanded across the Americas (Juliano 2010; Garcia-Rejon et al. 2021), effectively displacing *Ae. aegypti* from most U.S. cities (Lounibos et al. 2002; Camara et al. 2016). Both species inhabit artificial containers in the juvenile stages (egg, larvae, and pupae), corresponding to their close association with urban environments and human dwellings. The wider distribution of *Ae. albopictus* is attributed to its competitive superiority during this larval stage (Juliano 2010), given its ability to better exploit food resources within container habitats (Yee et al. 2004; Juliano 2010; Camara et al. 2016; Lounibos and Juliano 2018). The relative competitive inferiority of *Ae. aegypti* may be alleviated in some cities by its higher tolerance to heat and egg desiccation (Lounibos et al. 2002; Camara et al. 2016; Lounibos and Juliano 2018). In cities where both species are found, such as New Orleans, Louisiana, higher heat microenvironments, or urban heat islands (UHI), may help support the presence of *Ae. aegypti* (de Jesús Crespo and Rogers 2021). This interplay between competitive ability and tolerance to UHI patches may also allow for the coexistence of these species, rather than complete competitive exclusion (Camara et al. 2016). UHI and food resources within a city are closely linked to vegetation cover. Vegetation provides a cooling effect through shading and evapotranspiration, which consistently mitigates UHI (Li and Zhou 2019). Vegetation also provides leaf litter, seeds, insects, and other food sources that form a rich and diverse larval food base (Yee et al. 2015). High-quality food sources have been associated with higher adult mosquito fitness (Yee et al. 2015; Yan et al. 2021), which in turn, may influence susceptibility to pathogens (Carvajal-Lago et al. 2021). High quality larval food sources include insect carcasses and rapidly decaying plant detritus, while slowly-decaying plant detritus is considered a low quality larval food source (Fish and Carpenter 1982; Dieng et al. 2002). Containers with dense canopy cover may be more likely to contain high quality larval food sources from the canopy above, while containers with little to no canopy cover may rely more on autochthonous food sources such as algae (Rogers et al. 2023). The relative quality of this basal food relative to external (allochthonous) resources is not fully understood in container environments. Containers with an open canopy may be limited to the tolerant algae species which may be less nutritious and potentially toxic (Dhillon et al. 1982; Marten 1987, 2007) or they may contain highly nutritious diatoms, as often occurs in freshwater ecosystems (Guo et al. 2016). They may also contain dead insects and high-quality grass clippings (Murrell and Juliano 2008), but the input of these high quality resources would be expected to be more random and less frequent than for a container directly below a tree. Vegetation cover gradients within cities can therefore moderate microclimate and food web dynamics in container environments, with potential effects on mosquito vector assemblages and vector-borne disease risk.

In the context of *Ae. aegypti* and *Ae. albopictus* competition, vegetation-driven differences in microhabitat qualities may be relevant for human health. *Ae. albopictus*, which is associated with more vegetated and cooler habitats in cities where the species coexist (de Jesús Crespo and Rogers 2021), is also a less competent vector for at least one of the most prevalent mosquito-borne diseases, dengue fever. This is due to its lower infectivity rates to certain dengue virus serotypes (Whitehorn et al. 2015), and its higher frequency of feeding on non-human hosts (Lambrechts et al. 2010). If *Ae. albopictus* were to become infected with dengue, it is more likely to take the disease into other vertebrates, relative to *Ae. aegypti*, which preferentially seeks human hosts (Sivan et al. 2015). Although some studies have suggested that dengue can replicate in various vertebrate cell lines, no study has documented viral amplification of dengue in non-primate vertebrates in the wild (Hanley et al. 2013), making spillover back to humans unlikely, especially in places where native primates are nonexistent. It is also unlikely for non-primate amplification of chikungunya virus to occur (Vourc’h et al. 2014), as is the case for Zika virus (Weaver et al. 2016). Likewise, lower infectivity rates have been suggested for *Ae. albopictus* relative to *Ae. aegypti* for Zika virus (Lozano-Fuentes et al. 2019) as well as certain strains of chikungunya (Vega-Rúa et al. 2014).

Population dynamics of *Aedes* mosquitoes and resulting disease transmission has been previously studied from many different perspectives. Numerous works investigate the impacts of temperature on population dynamics of a single species and disease transmission. For instance, Erickson et al. explored the role of temperature dependence in development and mortality rates on the population dynamics of *Ae. albopictus* in a stage-structured differential equations model (Erickson et al. 2010a) as well as the implications for dengue transmission (Erickson et al. 2010b). Robert et al. (2019) incorporated temperature-dependent adult mortality and juvenile development rates into the population dynamics of *Ae. aegypti*, along with extrinsic incubation rates into a model of dengue to investigate the role of seasonality in dengue risk potential. Caldwell et al. (2021) incorporated temperature, along with rainfall and humidity, into *Ae. aegypti* and *Aedes*-borne arbovirus dynamics to understand the model’s predictive ability in two different countries. Other work includes multiple mosquito species, but no effects of competition, such as the work of Manore et al. (2014), in which they compared dengue and chikungunya transmission by *Ae. aegypti* and *Ae. albopictus*. Still other work includes competition, but does incorporate the impacts of temperature. Bewick et al. (2016) developed a temperature-independent model for La Crosse virus transmission incorporating competition between vectors *Ae. albopictus* and *Ae. triseriatus*, and Dimas Martins et al. (2022) developed a temperature-independent model for vector-borne disease incorporating competition between multiple hosts and vectors, exploring the role of ecological competition on the basic reproduction number $R_0$. In work combining competition and temperature-based models, Paton (2019) previously modeled competition between *Ae. aegypti* and *Ae. albopictus*, focusing on the role of reproductive interference, and Marini et al. (2017) modeled asymmetric interspecific competition between *Ae. albopictus* and *Culex pipiens*, incorporating temperature-dependent development and mortality rates.

Here, we develop a mechanistic model of competition between *Ae. albopictus* and *Ae. aegypti*, including juvenile and adult stages since competition occurs at the juvenile stage while disease transmission occurs at the adult stage. We incorporate differences in competition coefficients due to food resource quality as well as temperature dependence in life history parameters. We then explore the outcome of resulting species distributions on dengue transmission. We are further interested in exploring how vegetation-driven gradients in UHI and food quality could affect dengue fever risk, mediated by the spatial distribution of *Ae. albopictus* and *Ae. aegypti*. To our knowledge, this is the first study to evaluate how landscape factors that drive the habitat segregation patterns of different mosquito vectors varying in competence can contribute to disease dynamics at a neighborhood scale within a city.

In the sections that follow, we first develop a stage-structured ordinary differential equation model for *Ae. albopictus* and *Ae. aegypti* with competition between the juvenile stages. We incorporate experimental constraints on competition coefficients for low- and high-quality food and explore differences in the resulting outcomes of competition. We then allow fecundity, juvenile development rate, and adult mortality to be temperature-dependent and explore competition outcomes as a function of temperature. Finally, we investigate the combined impacts of temperature and competition on the potential for disease transmission and discuss our results in the context of present and future risk of mosquito-borne disease transmission.

## Mosquito Competition Model

We develop a stage-structured ordinary differential equation model for the population dynamics of *Ae. albopictus* and *Ae. aegypti* that includes one adult stage for each mosquito species along with one juvenile stage for each species that encompasses the egg, larval, and pupal life stages.

### Model Description

We let $J_i$ and $A_i$ represent the juvenile and adult classes, respectively, of species *i*, where $i=1$ represents *Ae. aegypti* and $i=2$ represents *Ae. albopictus*. The dynamics of the four populations $J_1$, $A_1$, $J_2$, and $A_2$ are governed by the following system of equations:

1 $$\begin{aligned} \frac{dJ_1}{dt}&= f_1A_1(1-\alpha _{11}J_1-\alpha _{12}J_2)- d_1J_1, \nonumber \\ \frac{dA_1}{dt}&= d_1J_1 - \mu _{1} A_1, \nonumber \\ \frac{dJ_2}{dt}&= f_2A_2(1-\alpha _{22}J_2-\alpha _{21}J_1)-d_2 J_2, \nonumber \\ \frac{dA_2}{dt}&= d_2J_2 - \mu _{2}A_2. \end{aligned}$$

The dynamics of each stage are taken as a difference of recruitment and mortality rates. New juveniles are born based on the per capita fecundity rate of each species, $f_i$, which represents the number of eggs per adult female per day that hatch into larvae and survive to become female adults in the absence of competition, implicitly accounting for density-independent juvenile mortality. We incorporate both interspecific and intraspecific larval competition between the two species in the juvenile equations; as in Bewick et al. (2016), we assume the presence of juveniles of either species reduces the number of larvae surviving to the adult stage, with the competition coefficients $\alpha _{ij}$ determining the competitive effect of species *j* on species *i*. We note the $\alpha _{ij}$ represent absolute competition coefficients, as in Juliano (2010) and Chesson (2000), rather than relative competition coefficients. We assume that adult mortality and juvenile development are density-independent with per capita adult mortality rate $\mu _i$ and per capita juvenile development rate $d_i$. The fecundity rate ($f_i$), juvenile development rate ($d_i$), and adult density-independent mortality rate ($\mu _i$) of each species may depend on temperature while the competition coefficients are assumed to be independent of temperature. All parameter values, together with brief descriptions, are listed in Table 1.

**Table 1:** Parameter descriptions for the stage-structured mosquito competition model (1), where $i=1$ represents *Ae. aegypti* and $i=2$ represents *Ae. albopictus*

Parameter	Description
$f_1$	Fecundity rate of *Ae. aegypti*
$f_2$	Fecundity rate of *Ae. albopictus*
$\alpha _{21}$	Competitive effect of *Ae. aegypti* on *Ae. albopictus*
$\alpha _{12}$	Competitive effect of *Ae. albopictus* on *Ae. aegypti*
$\alpha _{11}$	Competitive effect of *Ae. aegypti* on *Ae. aegypti*
$\alpha _{22}$	Competitive effect of *Ae. albopictus* on *Ae. albopictus*
$d_1$	Development rate of juvenile *Ae. aegypti*
$d_2$	Development rate of juvenile *Ae. albopictus*
$\mu _{1}$	Mortality rate of adult *Ae. aegypti*
$\mu _{2}$	Mortality rate of adult *Ae. albopictus*

### Equilibrium Stability Analysis

We obtain equilibrium values for System 1 by setting $\left( \dfrac{dJ_1}{dt}, \dfrac{dA_1}{dt}, \dfrac{dJ_2}{dt}, \dfrac{dA_2}{dt} \right) = \vec {0}$ and solving the resulting system for $(J_1^*,A_1^*,J_2^*,A_2^*)$. Model (1) has 4 equilibria, with one trivial equilibrium representing extinction of both species ($\mathscr {E}_1$), an equilibrium corresponding to persistence of *Ae. aegypti* and exclusion or extinction of *Ae. albopictus* ($\mathscr {E}_2$), another corresponding to persistence of *Ae. albopictus* and exclusion or extinction of *Ae. aegypti* ($\mathscr {E}_3$), and a fourth coexistence equilibrium where both species persist ($\mathscr {E}_4$). Expressions for each of the four equilibria are given here:

$$\begin{aligned} \mathscr {E}_1=&(0,0,0,0)\\ \mathscr {E}_2=&\left( \dfrac{f_1-\mu _1}{f_1\alpha _{11}}, \dfrac{d_1(f_1-\mu _1)}{\mu _1f_1\alpha _{11}}, 0, 0 \right) \\ \mathscr {E}_3=&\left( 0, 0, \dfrac{f_2-\mu _2}{f_2\alpha _{22}}, \dfrac{d_2(f_2-\mu _2)}{\mu _2f_2\alpha _{22}}\right) \\ \mathscr {E}_4=&\left( J_1^*, \frac{d_1}{\mu _1}J_1^*, J_2^*, \frac{d_2}{\mu _2}J_2^* \right) , \end{aligned}$$

where

$$\begin{aligned} J_1^* = \dfrac{f_1f_2(\alpha _{12}-\alpha _{22})-f_1\alpha _{12}\mu _2+f_2\alpha _{22}\mu _1}{f_1f_2(\alpha _{12}\alpha _{21}-\alpha _{11}\alpha _{22})} \end{aligned}$$

and

$$\begin{aligned} J_2^* = \dfrac{f_1f_2(\alpha _{21}-\alpha _{11})-f_2\alpha _{21}\mu _1+f_1\alpha _{11}\mu _2}{f_1f_2(\alpha _{12}\alpha _{21}-\alpha _{11}\alpha _{22})}. \end{aligned}$$

We note that the juvenile development rate affects only the equilibrium number of adults in the population; as the juvenile population transitions to adults at a faster rate and the time spent in the juvenile stages decreases, the equilibrium population size of adults increases.

The net reproduction number of each population in isolation is $n_i = \dfrac{f_i}{\mu _i}$. The extinction state $\mathscr {E}_1$ is stable when $\mu _1 > f_1$ ($n_1 < 1$) and $\mu _2 > f_2$ ($n_2 < 1$), as the condition for species *i* to persist in isolation is $\mu _i < f_i$ ($n_i > 1$). If $\mu _1 < f_1$ and $\mu _2 > f_2$, *Ae. aegypti* will persist while *Ae. albopictus* goes extinct ($\mathscr {E}_2$ stable), and if $\mu _1 > f_1$ and $\mu _2 < f_2$, *Ae. aegypti* will go extinct while *Ae. albopictus* persists ($\mathscr {E}_3$ stable). Assuming $\mu _1 < f_1$ and $\mu _2 < f_2$, so both species persist in isolation, then *Ae. aegypti* will competitively exclude *Ae. albopictus* if $\dfrac{\mu _2}{f_2} > 1 - \dfrac{\alpha _{21}}{\alpha _{11}}(1-\dfrac{\mu _1}{f_1})$ ($\mathscr {E}_2$ is stable). If $\dfrac{\mu _1}{f_1} > 1 - \dfrac{\alpha _{12}}{\alpha _{22}}(1-\dfrac{\mu _2}{f_2})$, then the equilibrium with *Ae. albopictus* competitively excluding *Ae. aegypti*, $\mathscr {E}_3$, is stable.

To simplify the discussion of stability in the context of competition parameters, we define $M_i=1-\dfrac{\mu _i}{f_i}$, $i=1,2$. Note there is a relationship between $M_i$ and the net reproduction number of the population, $n_i = \dfrac{f_i}{\mu _i}$, where $M_i = 1 - \dfrac{1}{n_i}$. $M_i$ is negative when $n_i < 1$ and increases with $n_i$ (which increases with $f_i$ and decreases with $\mu _i$), bounded above by 1. The species with the larger $n_i$ will have a larger value of $M_i$. We can rewrite the above conditions for stability in terms of $M_1$ and $M_2$. The extinction state, $\mathscr {E}_1$, is stable when $M_1 < 0$ and $M_2 < 0$. The *Ae. aegypti* only equilibrium, $\mathscr {E}_2$, is stable if $\dfrac{M_1}{M_2}>\dfrac{\alpha _{11}}{\alpha _{21}}$, and the *Ae. albopictus* only equilibrium, $\mathscr {E}_3$, is stable if $\dfrac{M_1}{M_2}<\dfrac{\alpha _{12}}{\alpha _{22}}$. Both single species equilibria, $\mathscr {E}_2$ and $\mathscr {E}_3$, are locally asymptotically stable when both criteria hold, with $\dfrac{\alpha _{11}}{\alpha _{21}}<\dfrac{M_1}{M_2}<\dfrac{\alpha _{12}}{\alpha _{22}}$. Here the outcome of competition depends on initial conditions. Note we must have $\dfrac{\alpha _{11}}{\alpha _{21}}<\dfrac{\alpha _{12}}{\alpha _{22}}$ for this to be possible. Both single species equilibria, $\mathscr {E}_2$ and $\mathscr {E}_3$, are unstable when $\dfrac{\alpha _{12}}{\alpha _{22}}<\dfrac{M_1}{M_2}<\dfrac{\alpha _{11}}{\alpha _{21}}$, resulting in coexistence of *Ae. aegypti* and *Ae. albopictus*. This outcome is only possible when $\dfrac{\alpha _{12}}{\alpha _{22}}<\dfrac{\alpha _{11}}{\alpha _{21}}$, or $\alpha _{12}\alpha _{21}<\alpha _{11}\alpha _{22}$. These results for the outcome of competition are summarized in Table 2.

If both species have equal fecundity and mortality rates, then $\dfrac{M_1}{M_2} = 1$ and conditions for coexistence reduce to that of the classical Lotka-Volterra model: $\alpha _{21}\alpha _{12} < \alpha _{11}\alpha _{22}$, or the product of the intraspecific competition coefficients must be greater than the product of the interspecific competition coefficients. $M_i$ can be increased by either increasing the fecundity rate or decreasing the mortality rate of species *i*. Therefore $\dfrac{M_1}{M_2}$ increases with increasing fecundity rate or decreasing mortality rate of species 1, or decreasing fecundity rate or increasing mortality rate of species 2. That is, changing parameters in a way that either benefits species 1 or harms species 2 will increase $\dfrac{M_1}{M_2}$. For given $\alpha _{ij}$, if $\dfrac{\alpha _{12}}{\alpha _{22}}<\dfrac{\alpha _{11}}{\alpha _{21}}$, increasing $\dfrac{M_1}{M_2}$ will result in the outcome of competition changing from *Ae. albopictus* excluding *Ae. aegypti* to coexistence to *Ae. aegypti* excluding *Ae. albopictus*. If $\dfrac{\alpha _{11}}{\alpha _{21}}<\dfrac{\alpha _{12}}{\alpha _{22}}$, then increasing $\frac{M_1}{M_2}$ will result in the outcome of competition changing from *Ae. albopictus* excluding *Ae. aegypti* directly to *Ae. aegypti* excluding *Ae. albopictus*, with the value of $\dfrac{M_1}{M_2}$ for which the switch occurs dependent upon the initial numbers of each species present.

In Section 3.1 we will explore how the ratio $\dfrac{M_1}{M_2}$, and consequently the outcome of competition, changes with temperature when mortality and fecundity rates are temperature dependent.

**Table 2:** Conditions for outcomes of competition of model (1), where $M_i=1-\dfrac{\mu _i}{f_i}>0$

Exclusion of *Ae. aegypti*	$\dfrac{M_1}{M_2}< \min \left\{ \dfrac{\alpha _{12}}{\alpha _{22}}, \dfrac{\alpha _{11}}{\alpha _{21}}\right\}$
Coexistence	$\dfrac{\alpha _{12}}{\alpha _{22}}<\dfrac{M_1}{M_2}<\dfrac{\alpha _{11}}{\alpha _{21}}$
Outcome depends on initial conditions	$\dfrac{\alpha _{11}}{\alpha _{21}}<\dfrac{M_1}{M_2}<\dfrac{\alpha _{12}}{\alpha _{22}}$
Exclusion of *Ae. albopictus*	$\max \left\{ \dfrac{\alpha _{12}}{\alpha _{22}}, \dfrac{\alpha _{11}}{\alpha _{21}}\right\} <\dfrac{M_1}{M_2}$

### Food Quality Dependent Competition Coefficients

Juliano (2010) conducted a meta-analysis of experiments looking at how increased densities of *Ae. aegypti* and *Ae. albopictus* affect survivorship to adulthood in order to determine the relative magnitudes of the absolute interspecific and intraspecific competition coefficients for the two species. He found the results to depend on the quality of the food source available. For low quality food sources, taken to be deciduous or coniferous tree leaf litter, the interspecific effect of *Ae. albopictus* was found to be greater than the intraspecific effect of *Ae. albopictus* ($\alpha _{12}>\alpha _{22}$), whereas the opposite was true for *Ae. aegypti* ($\alpha _{21}<\alpha _{11}$). Also, *Ae. albopictus* was more affected by intraspecific competition than interspecific competition ($\alpha _{21}<\alpha _{22}$) while the opposite was true for *Ae. aegypti* ($\alpha _{12}>\alpha _{11}$). Daugherty et al. (2000) found *Ae. albopictus* to have higher survivorship than *Ae. aegypti* in cultures with lower quality resources. Combining these results gives the following relationship between all competition coefficients for low quality food: $\alpha _{21}<\alpha _{11},\alpha _{22}<\alpha _{12}$. In the absence of data to characterize the relationship between $\alpha _{11}$ and $\alpha _{22}$ we assume they are equal.

For high quality food sources, taken to be dead insects, liver powder, yeast or grass, Juliano did not find the interspecific and intraspecific competitive effects to be significantly different (Juliano 2010) for either species. However, for *Ae. albopictus* the difference between the interspecific and intraspecific competitive effects for high quality food was significantly different from that seen for low quality food, while for *Ae. aegypti* there was no significant difference between high and low food quality. Thus we assume $\alpha _{21} \le \alpha _{11}$ and $\alpha _{12} \le \alpha _{22}$. Murrell and Juliano (2008) found neither species to be significantly affected by interspecific competition in high quality grass detritus cultures, and Daugherty et al. (2000) found the addition of high quality resource in the form of carcasses to improve survival for both species. We assume all $\alpha _{ij}$ decrease from low to high quality food, with $\alpha _{12}$ decreasing to be equal to $\alpha _{21}$, resulting in $\alpha _{21}=\alpha _{12} \le \alpha _{11}=\alpha _{22}$ for the high quality food scenario. The conditions on $\alpha _{ij}$ for low and high quality food are summarized in Table 3.

**Table 3:** Competition Coefficients. Assumed relationships between competition coefficients for model (1) of competition between *Ae. aegypti* and *Ae. albopictus*

Food Quality	Assumptions on Competition Coefficients
Low Quality Food	$\alpha _{21}< \alpha _{22} = \alpha _{11} < \alpha _{12}$
High Quality Food	$\alpha _{21}= \alpha _{12} \le \alpha _{11} = \alpha _{22}$

We note that scaling all competition coefficients by the same constant factor results in all equilibrium population sizes being divided by that factor, but does not affect the outcome of competition, which is dependent on the relative size of the $\alpha _{ij}$ rather than their magnitudes. Throughout this work we will consider three potential sets of $\alpha _{ij}$ - two sets (A and B) chosen to satisfy the assumptions for low quality food resources (one where coexistence is possible and one where coexistence is not possible) and one set chosen to satisfy the assumptions for high quality food resources. These values are given in Table 4. All competition coefficients are assumed to be independent of temperature.

**Table 4:** Assumed values of competition coefficients for low and high quality food scenario simulations

Competition Coefficient	Low Food Quality (A)	Low Food Quality (B)	High Food Quality
$\alpha _{11}$	$\dfrac{1}{60}$	$\dfrac{1}{60}$	$\dfrac{1}{100}$
$\alpha _{12}$	$\dfrac{1}{40}$	$\dfrac{1}{20}$	$\dfrac{1}{200}$
$\alpha _{21}$	$\dfrac{1}{180}$	$\dfrac{1}{90}$	$\dfrac{1}{200}$
$\alpha _{22}$	$\dfrac{1}{60}$	$\dfrac{1}{60}$	$\dfrac{1}{100}$
Ratio			
$\dfrac{\alpha _{11}}{\alpha _{21}}$	3	1.5	2
$\dfrac{\alpha _{12}}{\alpha _{22}}$	1.5	3	0.5

The criteria for which *Ae. albopictus* excludes *Ae. aegypti* can be written as $\mu _2 < f_2\left( 1-\dfrac{\alpha _{22}}{\alpha _{12}}\right) +\dfrac{f_2}{f_1}\dfrac{\alpha _{22}}{\alpha _{12}}\mu _1$. Once $\mu _2$ is large enough the *Ae. albopictus*-only equilibrium loses stability, and this threshold linearly increases with $\mu _1$. Similarly, the criteria for which *Ae. aegypti* excludes *Ae. albopictus* can be written as $\mu _2 > f_2\left( 1-\dfrac{\alpha _{21}}{\alpha _{11}}\right) +\dfrac{f_2}{f_1}\dfrac{\alpha _{21}}{\alpha _{11}}\mu _1$. Once $\mu _2$ is large enough the *Ae. aegypti* only equilibrium gains stability, and this threshold also linearly increases with $\mu _1$. The exclusion curves converge at $(\mu _1, \mu _2) = (f_1, f_2)$. For $\mu _1>f_1$ (resp. $\mu _2 > f_2$), *Ae. aegypti* (resp. *Ae. albopictus*) cannot persist in isolation.

**Fig. 1:**
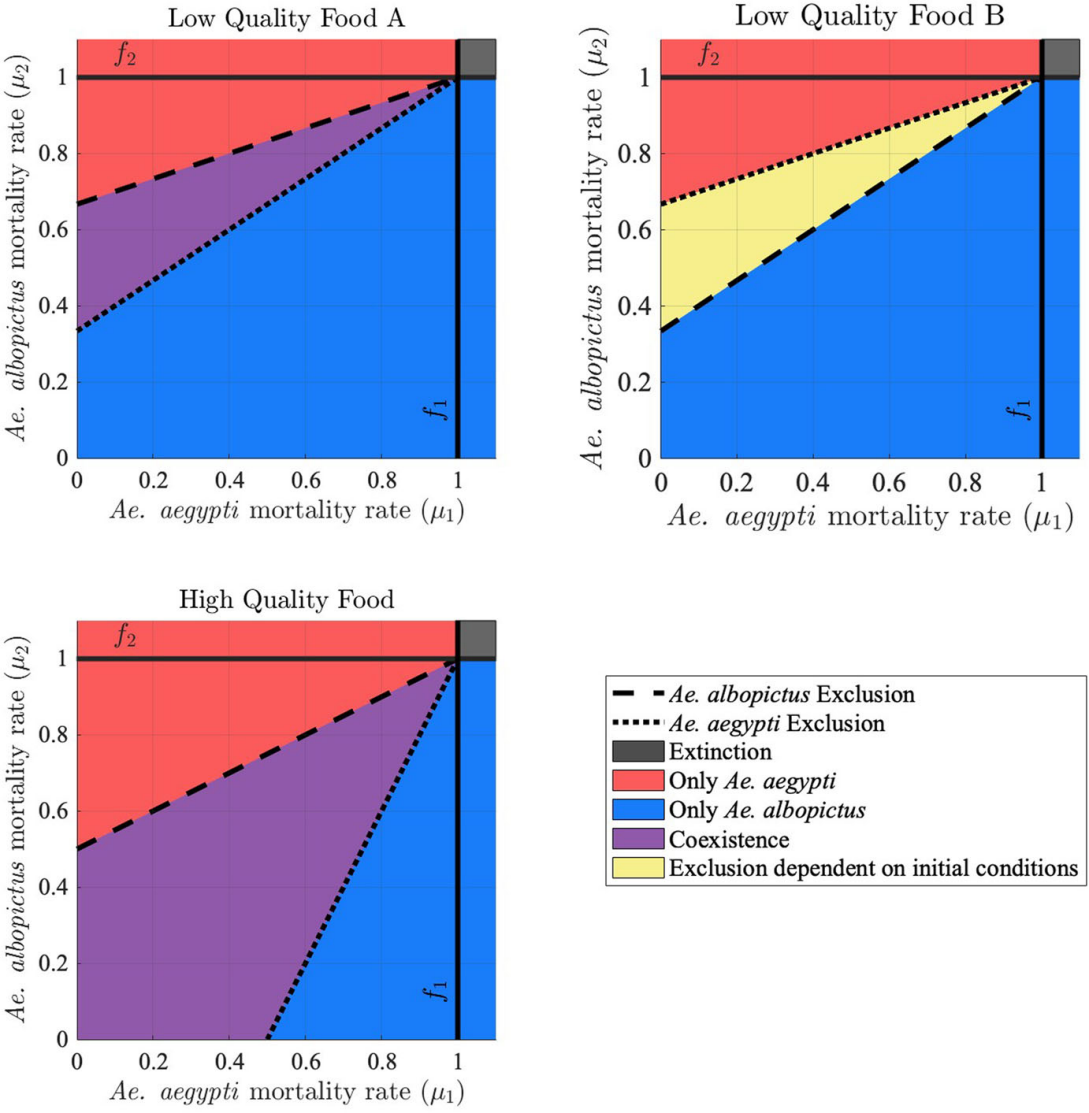
Outcomes of competition model (1) are shown as a function of mortality rates $\mu _1$ and $\mu _2$, for each of the three food quality assumptions and corresponding values of competition coefficients from Table 4. Fecundity rates $f_1=f_2=1$. The dashed line is the *Ae. albopictus* exclusion curve; *Ae. albopictus* cannot persist above this line. The dotted line is the *Ae. aegypti* exclusion curve; *Ae. aegypti* cannot persist below this line. If the *Ae. albopictus* exclusion curve is above the *Ae. aegypti* exclusion curve, we see coexistence as the outcome of competition in between the two lines (as for low quality food A and high quality food). If the *Ae. albopictus* exclusion curve lies below the *Ae. aegypti* exclusion curve, both exclusion equilibria are locally stable in between the two lines and the outcome of competition depends on initial conditions (as for low quality food B)

The exclusion thresholds for $\mu _2$ as a function of $\mu _1$, along with the outcomes of competition for all values of $\mu _1$ and $\mu _2$, are shown in Figure 1 for each of the three sets of competition coefficients (low food quality A and B, and high food quality) from Table 4. The fecundity rates of the two species are assumed to be equal with $f_1 = f_2 = 1$.

For low quality food, both exclusion curves have positive vertical intercepts, so equal adult mortality rates result in *Ae. albopictus* excluding *Ae. aegypti*; the mortality rate of *Ae. albopictus* must be greater than that of *Ae. aegypti* in order for *Ae. aegypti* to persist. The region of coexistence for scenario A is identical to the region where exclusion depends on initial conditions for scenario B, as $\dfrac{\alpha _{22}}{\alpha _{12}}$ for scenario A is equal to $\dfrac{\alpha _{21}}{\alpha _{11}}$ for scenario B and $\dfrac{\alpha _{21}}{\alpha _{11}}$ for scenario A is equal to $\dfrac{\alpha _{22}}{\alpha _{12}}$ for scenario B.

For high quality food, the vertical intercept of the *Ae. albopictus* exclusion curve is positive while the vertical intercept of the *Ae. aegypti* exclusion curve is negative, so equal adult mortality rates for the two species would result in coexistence. The region of coexistence is larger for high quality food compared to low quality food scenario A, with *Ae. aegypti* able to persist for a wider range of values of $\mu _1$ and $\mu _2$, including cases where the mortality rate of *Ae. aegypti* exceeds that of *Ae. albopictus*.

For high quality food, increasing intraspecific competition ($\alpha _{11}=\alpha _{22}$) or decreasing interspecific competition ($\alpha _{21}=\alpha _{12}$) while maintaining the conditions in Table 2 decreases the slope of the *Ae. albopictus* exclusion curve while increasing the vertical intercept and increases the slope of the *Ae. aegypti* exclusion curve while lowering the vertical intercept, thereby increasing the size of the region of coexistence.

The adult mortality rates of both species are temperature dependent, and the difference between them can also vary with temperature and affect the outcome of competition, as detailed in the next section and Section 3.1.

### Temperature-Dependent Parameters

**Fig. 2:**
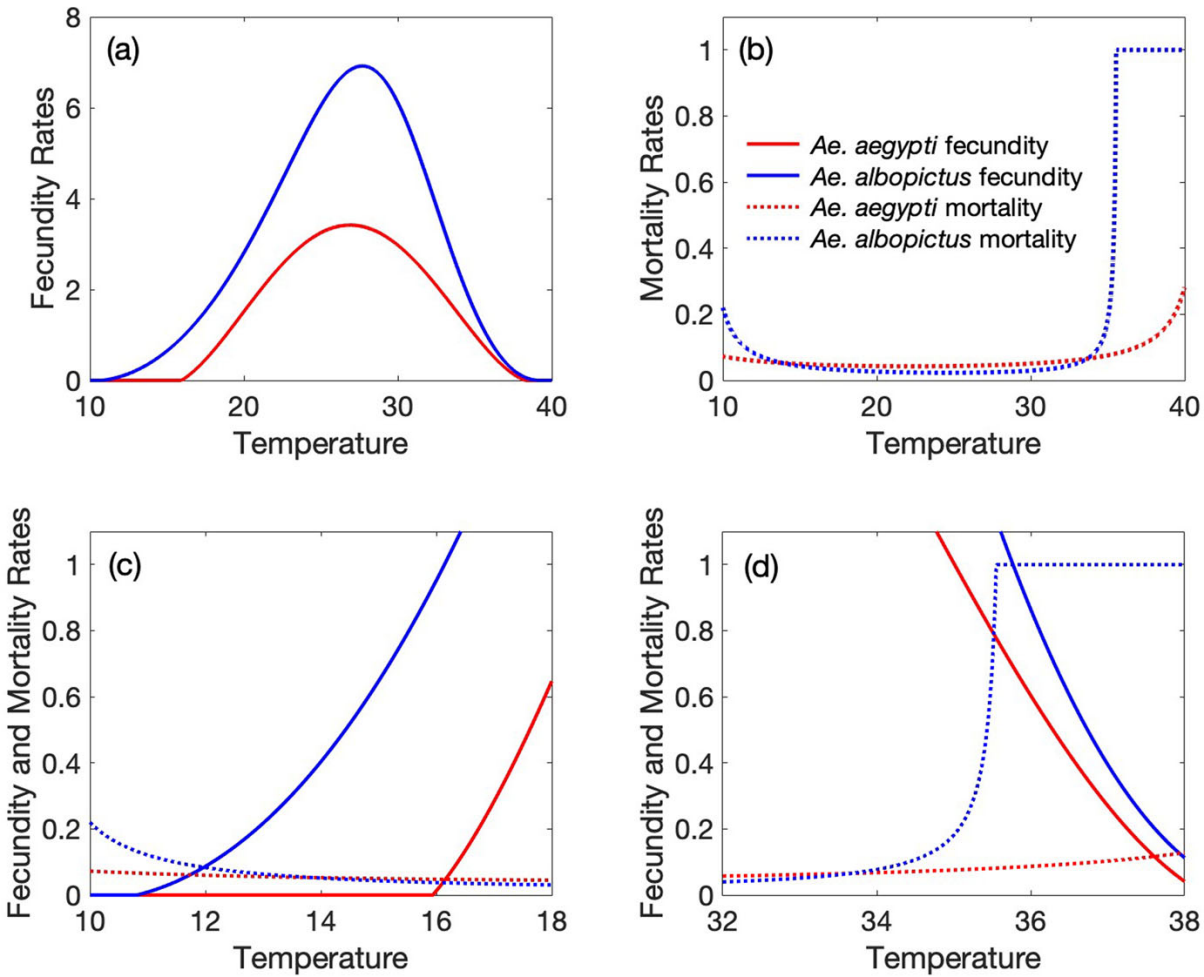
Shown are (a) fecundity rates (number of female eggs per adult female per day surviving to adulthood) and (b) adult mortality rates for each species as a function of temperature ($^\circ$C). Fecundity and mortality rates for the two species are shown together for temperatures between 10$^\circ$C and 18$^\circ$C in (c) and 32$^\circ$C and 38$^\circ$C in (d)

Temperature affects multiple aspects of the mosquito life cycle, including the length of the gonotrophic cycle; number of eggs laid per oviposition; the probability of eggs hatching and surviving to the larval, pupal, and adult stages; the development rate through these stages; and adult lifespan. We incorporate temperature dependence into the fecundity rates, $f_i$ (number of female eggs per female per day surviving to adulthood), juvenile-to-adult development rates, $d_i$, and adult mortality rates, $\mu _i$, of each species. Detailed descriptions and equations for each parameter as a function of temperature are given Appendix A.1. Plots of the fecundity and adult mortality rates of both species versus temperature are shown in Figure 2.

## Results

### Temperature and Competition

In order to explore the effects of temperature on the outcome of vector competition, we start by determining the values of $M_1$ and $M_2$ for temperatures between 10$^{\circ }$C and 40$^{\circ }$C. As $f_1$, $\mu _1$, $f_2$, and $\mu _2$ all change with temperature, so do $M_1$, $M_2$, and $\dfrac{M_1}{M_2}$.

**Fig. 3:**
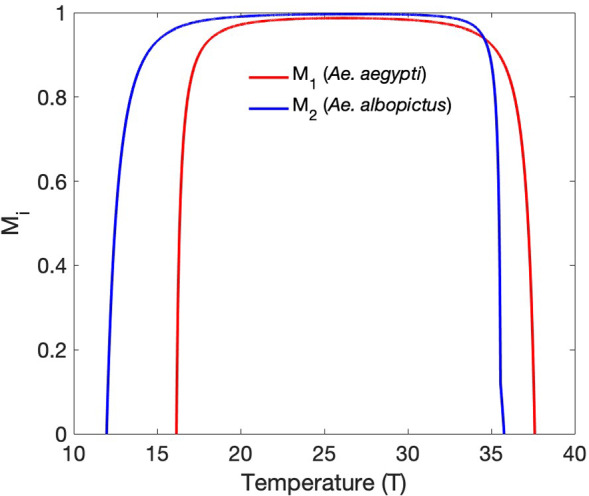
$M_i = 1 - \dfrac{\mu _i}{f_i}$ is shown as a function of temperature ($^{\circ }$C). Here, $M_1$ corresponds to *Ae. aegypti* and $M_2$ corresponds to *Ae. albopictus*

Figure 3 shows $M_1$ and $M_2$ versus temperature, where $M_i = 1 - \dfrac{\mu _i}{f_i}$. $M_1$ is positive ($\mu _1 < f_1$) for temperatures between 16.2$^{\circ }$C and 37.6$^{\circ }$C, while $M_2$ is positive ($\mu _2 < f_2$) for temperatures between 12$^{\circ }$C and 35.8$^{\circ }$C. Therefore, between 12$^{\circ }$C and 16.2$^{\circ }$C, *Ae. albopictus* will be able to persist in isolation while *Ae. aegypti* cannot, and between 35.8$^{\circ }$C and 37.6$^{\circ }$C, *Ae. aegypti* will be able to persist in isolation while *Ae. albopictus* cannot.

$M_1$ is less than $M_2$ for temperatures between 16.2$^{\circ }$C and 34.6$^{\circ }$C, resulting in a ratio $\dfrac{M_1}{M_2}$ less than one. For temperatures between 34.6$^{\circ }$C and 35.8$^{\circ }$C, $\dfrac{M_1}{M_2}$ is greater than one. Once temperatures reach 35.8$^{\circ }$C, $M_2$ reaches zero. $\mu _2$ exceeds $f_2$ for temperatures above 35.8$^{\circ }$C and *Ae. albopictus* is unable to persist in the absence of competition. For temperatures between 35.8$^{\circ }$C and 37.6$^{\circ }$C, however, *Ae. aegypti* is still able to persist. Once temperatures reach 37.6$^{\circ }$C, $M_1$ also hits zero and the extinction equilibrium gains stability.

**Fig. 4:**
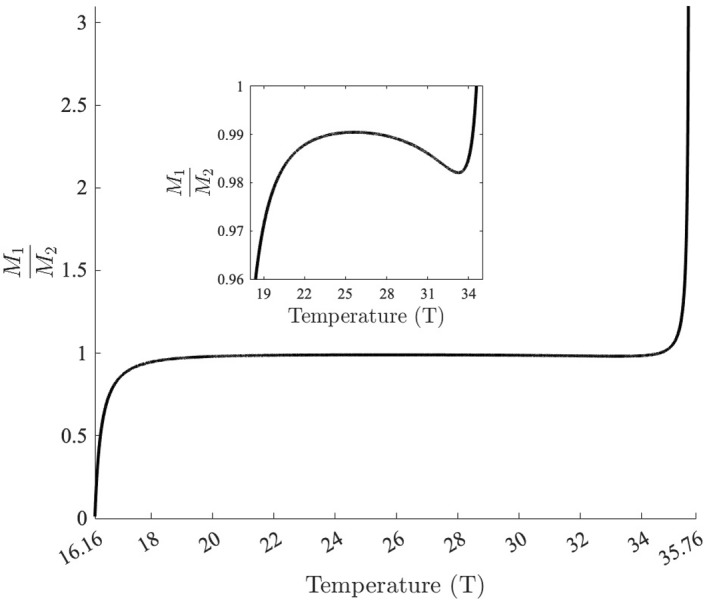
The ratio $\dfrac{M_1}{M_2}$ is shown as a function of temperature ($^{\circ }$C) over the range of temperatures where $M_1>0$ and $M_2 >0$. $M_1<0$ for $T<16.16$ and $M_2<0$ for $T > 35.76$. The magnitude of $\dfrac{M_1}{M_2}$ relative to $\dfrac{\alpha _{12}}{\alpha _{22}}$ and $\dfrac{\alpha _{11}}{\alpha _{21}}$ determines the outcome of competition between the two mosquito species. $\dfrac{M_1}{M_2}$ reaches 0.5 at $16.34^\circ$C , 1 at $34.57^\circ$C, 1.5 at $35.43^\circ$C, 2 at $35.49^\circ$C, and 3 at $35.52^\circ$C. (Inset) A closer look at the ratio $\dfrac{M_1}{M_2}$ between $19^\circ$C and $34^\circ$C. Note that $\dfrac{M_1}{M_2}$ is not monotonically increasing, but has a local minimum at $33.24^\circ$C

To further illustrate how the relationship between $M_1$ and $M_2$ changes with temperature, we show the ratio $\dfrac{M_1}{M_2}$ for different temperatures between 16.2$^{\circ }$C and 35.8$^{\circ }$C in Figure 4. Note that this is the range of temperatures for which both $M_1$ and $M_2$ are positive. As temperature increases from 16.16$^{\circ }$C, the ratio $\dfrac{M_1}{M_2}$ increases rapidly and is relatively constant, hovering just below 1, between about 19$^{\circ }$C and 34$^{\circ }$C. $\dfrac{M_1}{M_2}$ then decreases slightly reaching a local minimum at 33.24$^{\circ }$C before rapidly increasing again. This region where local extrema are present is important for the outcome of competition depending on values of $\alpha _{ij}$ as increasing temperature does not always lead to increases in the ratio $\dfrac{M_1}{M_2}$.

### The Impacts of Food Quality

**Fig. 5:**
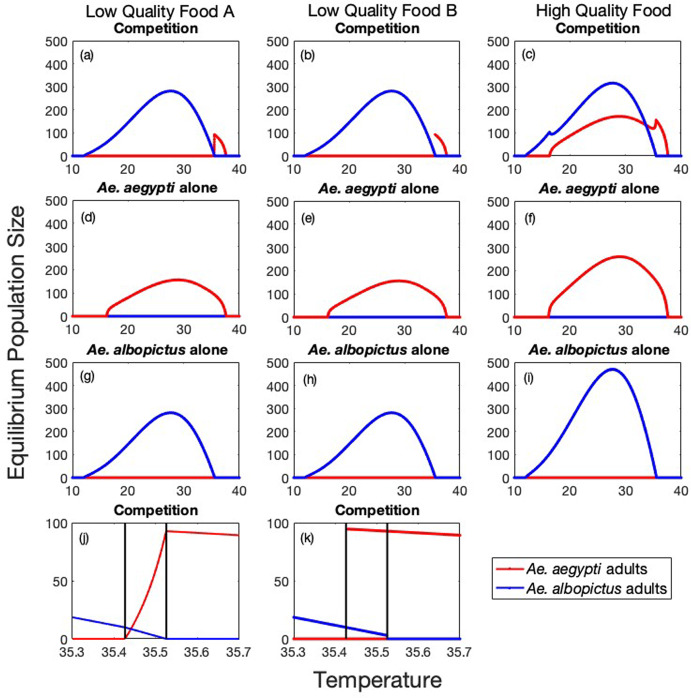
Equilibrium population sizes of *Ae. aegypti* and *Ae. albopictus* adults for low quality food A (a,d,g,j), low quality food B (b,e,h,k), and high quality food (c,f,i) parameter sets for competition coefficients as a function of temperature. Initial conditions for top row (a,b,c): $(J_1, A_1, J_2, A_2) = (0,100,0,100)$, second row (d,e,f): $(J_1, A_1, J_2, A_2) = (0,100,0,0)$ (*Ae. aegypti* only) and third row (g,h,i): $(J_1, A_1, J_2, A_2) = (0,0,0,100)$ (*Ae. albopictus* only). The fourth row is a zoomed in plot of the outcome of competition with both species present. Initial conditions for (j) are the same as in (a): $(J_1, A_1, J_2, A_2) = (0,100,0,100)$. Initial conditions for (k) are both $(J_1, A_1, J_2, A_2) = (0,1,0,1000)$ and $(J_1, A_1, J_2, A_2) = (0,1000,0,1)$. Vertical black lines are at T = 35.426 and T = 35.525, where $\frac{M_1}{M_2}$ equals 1.5 and 3, respectively. For this narrow range of temperatures ($35.426<T<35.525$), species coexist for low quality food A parameters (j), while for low quality food B parameters (k), the two exclusion equilibria are both locally stable with the outcome of competition depending on initial conditions (Color figure online)

We next investigated the impact of food quality on the outcome of competition and resulting equilibrium population sizes of the two vector species at different temperatures. We tested three different sets of competition coefficients - two different low quality food (A and B) and one high quality food scenario - to illustrate different outcomes. In Figure 5, we show equilibrium population sizes for the competition model (1) along with equilibrium population sizes for *Ae. aegypti* and *Ae. albopictus* in isolation for each set of competition coefficients.

In low quality food environments, we have from Table 3 that $\dfrac{\alpha _{12}}{\alpha _{22}}$ and $\dfrac{\alpha _{11}}{\alpha _{21}}$ are both greater than 1. This would always result in competitive exclusion of *Ae. aegypti* by *Ae. albopictus* if the fecundity and mortality rates of the two species were equal (i.e. $\dfrac{M_1}{M_2}=1$), or if *Ae. aegypti* either incurred greater mortality or had lower fecundity than *Ae. albopictus* (i.e. $\dfrac{M_1}{M_2}<1$). From Figure 4, $\dfrac{M_1}{M_2}$ is less than one until 34.6$^{\circ }$C, ensuring *Ae. albopictus* excludes *Ae. aegypti* for temperatures below 34.6$^{\circ }$C.

For temperatures between 34.6$^{\circ }$C and 35.8$^{\circ }$C, $\dfrac{M_1}{M_2}$ is greater than one and coexistence may be possible if $\dfrac{M_1}{M_2}$ exceeds $\dfrac{\alpha _{12}}{\alpha _{22}}$, with *Ae. aegypti* persisting alone if $\dfrac{M_1}{M_2}$ also exceeds $\dfrac{\alpha _{11}}{\alpha _{21}}$.

From Table 3, we cannot determine the relative magnitude of $\dfrac{\alpha _{12}}{\alpha _{22}}$ and $\dfrac{\alpha _{11}}{\alpha _{21}}$, but once $\dfrac{M_1}{M_2}$ exceeds the smaller of the two ratios the outcome of competition will change to either coexistence (if $\dfrac{\alpha _{12}}{\alpha _{22}}$ is smaller) or the outcome dependent on initial conditions (if $\dfrac{\alpha _{11}}{\alpha _{21}}$ is smaller). For low quality food scenario A, the smaller ratio is $\dfrac{\alpha _{12}}{\alpha _{22}}=1.5$ and for low quality food scenario B, the smaller ratio is $\dfrac{\alpha _{11}}{\alpha _{21}}=1.5$. $\dfrac{M_1}{M_2}$ reaches 1.5 at 35.43$^{\circ }$C, so at this temperature we see the outcome of competition change from *Ae. albopictus* excluding *Ae. aegypti* to coexistence for low quality food scenario A, and to exclusion of one species depending on initial conditions for low quality food scenario B. For low quality food scenario A, the larger ratio is $\dfrac{\alpha _{11}}{\alpha _{21}}=3$ and for low quality food scenario B, the larger ratio is $\dfrac{\alpha _{12}}{\alpha _{22}}=3$. $\dfrac{M_1}{M_2}$ reaches 3 at 35.52$^{\circ }$C, and we see the outcome of competition change to *Ae. aegypti* excluding *Ae. albopictus* for both low quality food scenarios for temperatures above 35.52$^{\circ }$C.

In high quality food environments, we have $\dfrac{\alpha _{12}}{\alpha _{22}}\le 1$ and $\dfrac{\alpha _{11}}{\alpha _{21}}\ge 1$ from Table 3. Therefore when species have equal fecundity and mortality rates, so $\dfrac{M_1}{M_2}=1$, the outcome of competition would be coexistence of *Ae. aegypti* and *Ae. albopictus*. Increasing the mortality of either species high enough or decreasing the fecundity of a species low enough would result in the exclusion of that species, but both exclusion equilibria will never be stable simultaneously, and the outcome of competition will never depend on initial conditions. For the high quality food parameter values in Table 4, $\dfrac{M_1}{M_2}$ exceeds the lower ratio of $\dfrac{\alpha _{12}}{\alpha _{22}}=0.5$ at 16.34$^{\circ }$C and the larger ratio of 2 at 35.49$^{\circ }$C, resulting in competitive exclusion of *Ae. aegypti* for temperatures between 16.16$^{\circ }$C (below which *Ae. aegypti* cannot persist in isolation) and 16.34$^{\circ }$C, coexistence between 16.34$^{\circ }$C and 35.49$^{\circ }$C, and competitive exclusion of *Ae. albopictus* between 35.49$^{\circ }$C and 35.8$^{\circ }$C. Above 35.8 degrees *Ae. albopictus* cannot persist in isolation and we see *Ae. aegypti* persist alone until 37.6$^{\circ }$C. For higher temperatures neither species can survive.

## Vector-borne Disease Transmission Model

We next explore how the outcome of competition between *Ae. aegypti* and *Ae. albopictus*, influenced by both food quality and temperature, in turn impacts the transmission of vector-borne diseases such as dengue, spread between adult mosquitoes and humans. To model disease transmission, we use an ordinary differential equation host-vector model with human hosts and two vector species (*Ae. aegypti* and *Ae. albopictus*) whose population dynamics are described by (1). We assume all mosquitoes are born susceptible and adult female mosquitoes move from the susceptible to the exposed class of their respective species upon biting an infectious host. They then progress to the infectious class at a rate determined by the extrinsic incubation period of the disease. We assume vectors remain infectious for life because there is a lack of evidence that either *Ae. aegypti* or *Ae. albopictus* recover within their lifespans. For the human hosts, we assume equal birth and death rates to maintain a constant population size, with all births into the susceptible class. A susceptible host moves to the exposed class upon being bitten by an infectious mosquito of either species, and then progresses to the infectious class at a rate determined by the intrinsic incubation period of the virus. We assume infectious hosts can recover with permanent immunity.

**Table 5:** Parameters for the disease transmission model (2)

Parameter	Description
$N_h$	Total human host population
$a_i$	Biting rate of species *i* on human hosts
$\rho _{v_i}$	Probability of transmission from humans to species *i*
$\rho _{h_i}$	Probability of transmission from species *i* to humans
$\sigma _{v_i}$	Extrinsic incubation rate of disease in species *i*
$\sigma _{h}$	Intrinsic incubation rate of disease in humans
$r_h$	Human recovery rate
$\mu _h$	Birth/death rate of humans

**Fig. 6:**
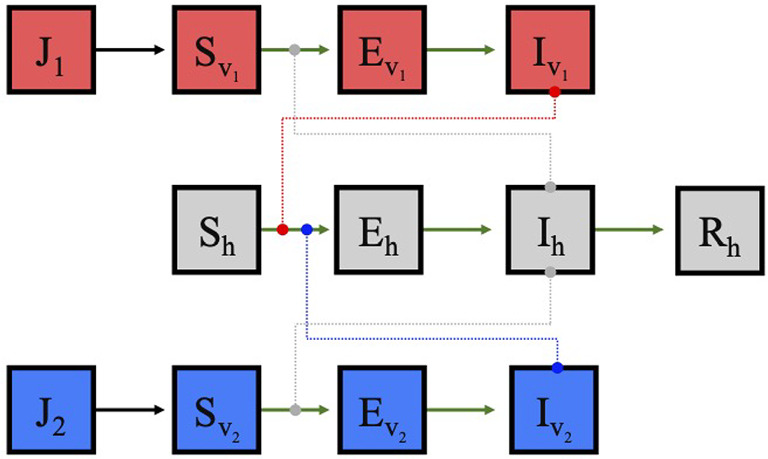
Schematic of model 2 for vector-borne disease transmission between two mosquito species and human hosts. Transmission occurs when infected hosts are bitten by susceptible adults of either mosquito species, or if infectious adults of either mosquito species bites a susceptible human host

A schematic of the stage-structured competition model incorporating disease transmission is shown in Figure 6 and the equations are as follows with parameters defined in Table 5:

2 $$\begin{aligned} \frac{dJ_1}{dt}= &  f_1N_{v_1}(1-\alpha _{11}J_1-\alpha _{12}J_2)-d_1J_1\nonumber \\ \frac{dS_{v_1}}{dt}= &  d_1J_1-\left( \frac{a_1\rho _{v_1}I_h}{N_h}\right) S_{v_1}-\mu _1 S_{v_1} \nonumber \\ \frac{dE_{v_1}}{dt}= &  \left( \frac{a_1\rho _{v_1}I_h}{N_h}\right) S_{v_1}-\sigma _{v_1}E_{v_1}-\mu _1E_{v_1} \nonumber \\ \frac{dI_{v_1}}{dt}= &  \sigma _{v_1}E_{v_1}-\mu _1I_{v_1} \nonumber \\ \frac{dJ_2}{dt}= &  f_2N_{v_2}(1-\alpha _{22}J_2-\alpha _{21}J_1)-d_2J_2 \\ \frac{dS_{v_2}}{dt}= &  d_2J_2-\left( \frac{a_2\rho _{v_2}I_h}{N_h}\right) S_{v_2}-\mu _2S_{v_2} \nonumber \\ \frac{dE_{v_2}}{dt}= &  \left( \frac{a_2\rho _{v_2}I_h}{N_h}\right) S_{v_2}-\sigma _{v_2}E_{v_2}-\mu _2E_{v_2} \nonumber \\ \frac{dI_{v_2}}{dt}= &  \sigma _{v_2}E_{v_2}-\mu _2I_{v_2} \nonumber \\ \frac{dS_h}{dt}= &  \mu _h N_h-\left( \frac{a_1\rho _{h_1}I_{v_1}+a_2\rho _{h_2}I_{v_2}}{N_h}\right) S_h-\mu _h S_h \nonumber \\ \frac{dE_h}{dt}= &  \left( \frac{a_1\rho _{h_1}I_{v_1}+a_2\rho _{h_2}I_{v_2}}{N_h}\right) S_h-\sigma _hE_h-\mu _h E_h \nonumber \\ \frac{dI_h}{dt}= &  \sigma _hE_h-r_hI_h-\mu _h I_h \nonumber \\ \frac{dR_h}{dt}= &  r_h I_h-\mu _h R_h\nonumber \end{aligned}$$

where $N_{v1} = S_{v1} + E_{v1} + I_{v1} \text { and } N_{v2} = S_{v2} + E_{v2} + I_{v2}$. We characterize disease transmission risk using $R_0$, the basic reproductive number, which is defined as the expected number of secondary cases that arise from a single infectious individual in a completely susceptible population. We calculate $R_0$ for our model using the next-generation matrix approach (Diekmann et al. 2010; Van den Driessche and Watmough 2002):

3 $$\begin{aligned} R_0=\sqrt{\frac{a_1\rho _{h_1}\sigma _{v_1}}{\mu _1(\mu _1+\sigma _{v_1})}\cdot \frac{a_1\rho _{v_1}\sigma _h N_{v_1}^*}{(\mu _h+r_h)(\mu _h+\sigma _h)N_h}+\frac{a_2\rho _{h_2}\sigma _{v2}}{\mu _2(\mu _2+\sigma _{v_2})}\cdot \frac{a_2\rho _{v2}\sigma _hN_{v_2}^*}{(\mu _h+r_h)(\mu _h+\sigma _h)N_h}}. \end{aligned}$$

We remark that $R_0$ increases with the vector-host ratios $\dfrac{N_{v1}^*}{N_h}$ and $\dfrac{N_{v2}^*}{N_h}$, where $N_{v1}^*$, $N_{v2}^*$, and $N_h$ represent the population sizes of adult *Ae. aegypti*, *Ae. albopictus*, and humans at the disease free equilibrium. In our calculations of $R_0$, $N_{v1}^*$ and $N_{v2}^*$ are determined by the vector competition model (1), and depend on the parameters $f_1$, $f_2$, $\mu _1$, $\mu _2$, $d_1$, and $d_2$, which all depend on temperature. We assume a human population size $N_h = 200$ for the results to follow in order to obtain $R_0$ values in a reasonable range (Liu et al. 2020). In addition to the temperature-dependent parameters affecting vector population dynamics, the disease parameters for vector biting rates, vector-to-host and host-to-vector transmission probabilities, and extrinsic incubation rates are also assumed to vary with temperature. Details for these parameters are given in Appendix A.2.

**Fig. 7:**
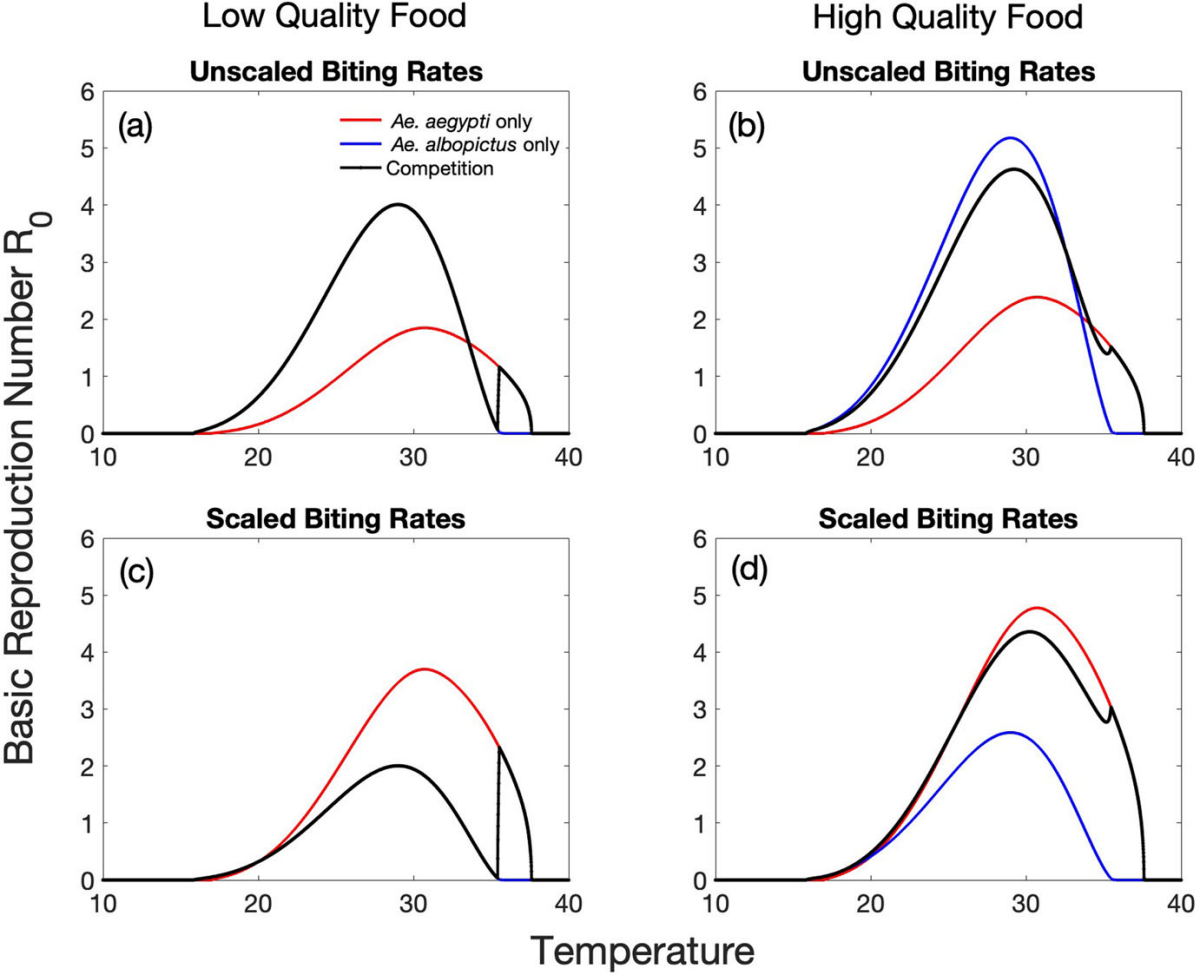
The basic reproduction number $R_0$ for model (2) is shown as a function of temperature ($^\circ$C) for low quality food scenario A parameters in the left column (a,c) and high quality food parameters in the right column (b,d). $R_0$ is computed for the vector equilibrium population sizes resulting from three different sets of initial conditions as shown in Figure 5 - *Ae. aegypti* alone (red), *Ae. albopictus* alone (blue), and both species present (black). The results in the top row (a,b) are for unscaled biting rates (16) and (17), with no multipliers on biting rates. The results in the bottom row (c,d) are for scaled biting rates (18) and (19), assuming a human bite multiplier of 0.5 for *Ae. albopictus* and 2 for *Ae. aegypti*. Note that when the outcome of competition is competitive exclusion of one species, the black curve coincides with either the blue or red curve, depending on which species persists (Color figure online)

We consider two different scenarios for vector biting rates. First, we take the biting rates to be the inverse of the gonotrophic cycle length as given in Mordecai et al. (2017). We refer to these as unscaled biting rates. Second, to obtain what we call scaled biting rates, we multiply these functions by a factor of 2 for *Ae. aegypti* and 0.5 for *Ae. albopictus* to account for potential multiple bites on humans per gonotrophic cycle by *Ae. aegypti* (Rezza 2012) and the tendency of *Ae. albopictus* to bite non-human hosts (Lambrechts et al. 2010). The basic reproduction number $R_0$ is shown as a function of temperature in Figure 7 for low quality food A and high quality food competition coefficients, and for both unscaled and scaled vector biting rates. For each combination of food quality and biting rate assumptions, $R_0$ is computed for the vector disease free equilibrium values resulting from competition as well as each species in isolation, as shown in Figure 5. We do not show results for low quality food scenario B competition coefficients; the outcome of competition (and therefore also $R_0$) for low quality food scenario B is always identical to that of low quality food scenario A for temperatures below 35.43$^\circ$C and above 35.52$^\circ$C, and equal to one of the single species equilibria (and corresponding $R_0$) in between. Coexistence is never possible for low food quality scenario B, and the $R_0$ curves for the species in isolation are identical to those for low food quality scenario A, since intraspecific competition coefficients $\alpha _{11}$ and $\alpha _{22}$ are the same.

At any given temperature, we find $R_0$ values to be higher for high quality food than for low quality food. This is a direct result of mosquito population sizes being higher for high quality food. In isolation, each species benefits from increased food quality with increased population sizes. With competition, *Ae. albopictus* numbers are similar for low and high food quality competition coefficients, but with high quality food *Ae. aegypti* is able to persist as well for a large range of temperatures, leading to higher overall vector population sizes and higher disease risk. However, $R_0$ is not solely determined by population sizes. Equal population sizes of the same species at different temperatures can result in a different value of $R_0$ as other components of $R_0$ are temperature-dependent.

For low quality food and unscaled biting rates, disease risk is maximized at $29^\circ$C with $R_0 = 4$, when *Ae. albopictus* excludes *Ae. aegypti* at an abundance of 274. When assuming scaled biting rates, the maximum disease risk changes to $R_0 = 2.3$ at $35.53^\circ$C, when *Ae. aegypti* excludes *Ae. albopictus* at a lower equilibrium population size of 92.6. We note this is higher than $R_0$ for high quality food at slightly higher temperatures ($36.5^\circ$C). Therefore if a high quality food environment experiences higher temperatures than a low quality food environment, it is possible disease risk may be higher in the low quality food environment.

For low quality food, disease risk is locally minimized at $35.42^\circ$C for both biting rate scenarios, when $\dfrac{M_1}{M_2}$ reaches 1.5 and the outcome of competition changes, allowing *Ae. aegypti* to persist above this temperature. Just below $35.42^\circ$C *Ae. albopictus* is still able to exclude *Ae. aegypti*, but at a low equilibrium population size. *Ae. albopictus* excludes *Ae. aegypti* with a population size of 87 at $34.22^\circ$C, and *Ae. aegypti* excludes *Ae. albopictus* with the same population size at $35.8^\circ$C. The corresponding $R_0$ values are higher when *Ae. aegypti* is present; $R_0$ is 1 at $34.22^\circ$C and 1.09 at $35.8^\circ$C for unscaled biting rates, and 0.5 at $34.22^\circ$C and 2.18 at $35.8^\circ$C for scaled biting rates.

When biting rates are scaled, the disease risk is always greater for populations of *Ae. aegypti* in isolation versus *Ae. albopictus* regardless of food quality (except at very low temperatures when $R_0$ is below 1). With unscaled biting rates, *Ae. albopictus* populations alone result in higher disease risk (compared to *Ae. aegypti* alone) for temperatures below $33.57^\circ$C; above this temperature $R_0$ is higher for populations of *Ae. aegypti* alone than *Ae. albopictus* alone.

For high quality food, there is a large range of temperatures resulting in coexistence of both species and overall maximum disease risk is similar for both biting rate assumptions; $R_0$ is maximized at 4.63 at $29.2^\circ$C for unscaled biting rates, and 4.36 at $30.3^\circ$C for scaled biting rates. At higher temperatures, $R_0$ is higher when biting rates are scaled, accounting for additional human bites per gonotrophic cycle for *Ae. aegypti* and bites on alternative hosts for *Ae. albopictus*. At $35^\circ$C, $R_0 = 2.8$ for scaled biting rates, while for unscaled biting rates disease risk is lower with $R_0 = 1.4$. When $R_0$ drops below 1 for unscaled biting rates at $36.77^\circ$C, $R_0=2$ at $36.77^\circ$C for scaled biting rates and does not drop below 1 until $37.44^\circ$C.

For high quality food and unscaled biting rates, populations of *Ae. albopictus* and *Ae. aegypti* resulting from competition are equal (131.6) at 33.7$^\circ$C, leading to an $R_0$ of 2.25, which is higher than $R_0$ for either species in isolation at the same temperature (*Ae. albopictus* reaches a population size of 194.1 with $R_0$ of 1.89 and *Ae. aegypti* reaches a population size of 203.1 with $R_0$ of 2.01). When biting rate differences in the mosquito species are incorporated, $R_0$ for *Ae. albopictus* in isolation is 0.94 at this temperature, while $R_0$ for *Ae. aegypti* is 4.03, over 4 times larger. The coexistence $R_0$ is 3.35, in between the $R_0$ values for the two species in isolation.

## Discussion

In this paper, we investigated competitive interactions between the mosquito species *Ae. aegypti* and *Ae. albopictus*, exploring the influence of temperature and food quality on the outcome of competition and the resulting risk of vector-borne disease transmission. We found the population dynamics of the two mosquitoes to depend on temperature and the relative strengths of inter- and intra-specific competition, which in turn depend on food quality. Taken together, these results highlight the importance of temperature and food quality on the relative abundance of these two species and the role of competition in the risk to humans of vector-borne diseases that these mosquitoes spread.

We found that high quality food can lead to coexistence of the two species across a range of temperatures where low quality food does not. When two species have similar fecundity and mortality rates, the quality of food available can impact the strength of intra- and inter-specific competition and determine whether the two species successfully coexist or whether one excludes the other.

Differences in mortality rates and fecundity rates between the two species also influence the outcome of competition. Increasing fecundity rates or decreasing mortality rates for one species will promote the persistence of that species, with the outcome of competition dependent on the differences in mortality and fecundity between the two species along with the strength of intra- and inter-specific competition determined by food quality.

Two species with equal fecundity and mortality rates will coexist for high quality food but not for low quality food. Increasing the mortality of one species high enough will eventually result in exclusion of that species regardless of food quality. If the mortality of two species are differentially affected by increasing temperature, we can also see a change in the outcome of competition. If *Ae. albopictus* suffers increased mortality due to heat compared to *Ae. aegypti*, we can see *Ae. aegypti* competitively exclude *Ae. albopictus* at high temperatures even when *Ae. albopictus* would still be able to persist in isolation.

Incorporating temperature dependence into model parameters, we see *Ae. albopictus* has an advantage in both fecundity rate and mortality rate at moderate temperatures. Since any set of competition coefficients satisfying the requirements for low quality food results in a necessary condition of $\dfrac{M_1}{M_2} > 1$ for coexistence, the temperature range for possible coexistence is limited to the narrow range of higher temperatures for which $\dfrac{M_1}{M_2}$ increases quickly. However, different choices of parameters may increase this range of possible coexistence.

Although *Ae. albopictus* may out-compete *Ae. aegypti* for a large range of temperatures in low quality food environments, *Ae. aegypti* is likely to persist at higher temperatures when *Ae. albopictus* cannot, regardless of food quality. Given its ability to survive higher temperatures, *Ae. aegypti* could become the dominant species in regions where average temperatures are warmest. Given that *Ae. aegypti* feeds more preferentially on humans than *Ae. albopictus*, a shift in competition that leads to *Ae. aegypti* populations excluding *Ae. albopictus* populations would have important impacts for mosquito-borne disease spread. This could lead to greater incidence of diseases such as dengue, Zika, and chikungunya in regions where temperatures are getting warmer due to climate change.

We found that estimates of $R_0$ varied by temperature, whether one or both species were present, and how biting rates were characterized. In some cases, lower populations of *Ae. aegypti* could lead to higher $R_0$ values than similar scenarios with higher populations of *Ae. albopictus*, in particular if *Ae. aegypti* bites on humans greatly outnumber those of *Ae. albopictus*. In low quality food environments, *Ae. albopictus* may suppress *Ae. aegypti* populations at low and moderate temperatures, leading to lower $R_0$ values than would be observed if *Ae. aegypti* alone were present. Once temperatures are high enough for *Ae. aegypti* to persist we would see a sharp increase in $R_0$.

Our results for $R_0$ and disease risk depend strongly on the assumptions regarding vector biting rates on humans. *Ae. aegypti* typically has a high biting rate on humans, often biting multiple times per gonotrophic cycle (Rezza 2012). *Ae. albopictus* may also bite humans at a high rate, but it depends on the availability of alternative hosts (Lambrechts et al. 2010). The probability of host-to-vector and vector-to-host transmission curves used here are similar at moderate temperatures for both species. However, there is evidence to suggest *Ae. aegypti* may have higher transmission probabilities (Vazeille et al. 2003; Chen et al. 1993). *Ae. albopictus* data may also be misleading depending on if the midgut or saliva data is used for infection rates (Whitehorn et al. 2015).

Our results have important implications for mosquito and arbovirus distribution in regions where both *Aedes* species can be found. For example, New Orleans consists of many regions with different microclimates on smaller spatial scales, varying in temperature, food quality, and human population density (de Jesús Crespo and Rogers 2021). Around $35^\circ$C, small differences in temperature between urban heat islands and more vegetated parts of the city can result in differences in competition outcomes which results in differences in the abundance of each species, particularly when food quality is low. These differences, in turn, lead to large differences in $R_0$ estimates. Thus, regions with heterogeneity in microclimates will have heterogeneous risk for disease transmission that will be driven by combinations of temperature, mosquito abundance, and quality of available food sources. This is important to consider when developing mitigation strategies and predicting impacts of climate on current and future mosquito abundance and disease risk.

Our model makes a number of simplifying assumptions. In order to focus on the effects of inter- and intra-specific competition, we assume there is no density-independent mortality in the larval stages. While this allows us to better tie our results to relationships between competition coefficients, it is worth noting that density-independent mortality is likely to affect population size, and significant differences between larval mortality of the two species could have impacts on competition. Furthermore, we focus on relative relationships between intra- and inter-specific competition. The magnitude of the differences in competition coefficients could vary widely, and we have characterized the differences in competition based on literature available; however, future studies of competition could improve model parameter estimates and facilitate a better understanding of how differences in competition impact populations. Finally, we consider the impacts of constant temperature throughout this study, focusing on equilibrium dynamics. Temperatures vary across days and seasons, and this variation in temperature will have an impact on mosquito population size, life history characteristics, competition, and disease transmission. A logical next step would be to expand on the current model by allowing for seasonal and/or diurnal variation in temperature and understanding how this variability could impact population dynamics and disease transmission.

Our work illustrates the importance of considering competition in models for population dynamics of *Ae. albopictus* and *Ae. aegypti* in regions where their distributions overlap. This is particularly critical when studying populations where temperature ranges could support both coexistence and competitive exclusion as well as regions currently on the margins of such ranges that could be impacted by increases in temperatures caused by climate change. Including competition, temperature dependence, and interactions between the two in models aimed at predicting future populations of both species will contribute to a better understanding of how distributions of each species will change, which will better guide mosquito surveillance and control efforts and improve our ability to predict areas at risk of future dengue transmission.

## Data Availability

No datasets were generated or analyzed in this work. MATLAB code used to generate figures is available upon request.

## Temperature-Dependent Parameters

### Competition Model Parameters

**Fecundity rates**

To determine the temperature dependent fecundity rate of *Ae. aegypti*, we first fit a quadratic curve to the data points for oviposition rate from Mordecai et al. (2017) between 15$^\circ$C and 35$^\circ$C. For *Ae. aegypti* the oviposition rate was given as number of eggs per day per female so we divided by 2 to obtain the number of female eggs per day per female:

4 $$\begin{aligned} \lambda _1(T) = \dfrac{\max (-0.0538T^2 + 2.99T -33.99,0)}{2} \end{aligned}$$

For *Ae. aegypti* egg to adult survival, we define (Mordecai et al. 2017):

5 $$\begin{aligned} p_1^{EA}(T) = \max (-.00599(T-38.29)(T-13.56),0) \end{aligned}$$

Then $$f_1 = \lambda _1(T)p_1^{EA}$$.

For *Ae. albopictus* the oviposition rate in Mordecai et al. (2017) was given as number of eggs per gonotrophic cycle per female:

6 $$\begin{aligned} \text {EGC}_2(T) = \max (-.3379T^2+16.86T-142.8,0). \end{aligned}$$

We then fit a quadratic curve to the duration of the gonotrophic cycle from Delatte et al. (2009):

7 $$\begin{aligned} \text {GCD}_2(T) = \max (0.045T^2 - 2.717T +44.41,0). \end{aligned}$$

Finally, we used the probability of egg to adult survival from Mordecai et al. (2017):

8 $$\begin{aligned} p_2^{EA}(T) = \max (-.00361(T-39.33)(T-9.04),0). \end{aligned}$$

We then divided the product of the oviposition rate (6) and egg to adult survival rate (8) by 2 times the duration of the gonotrophic cycle (7) to obtain the number of female eggs per day per female, $f_2(T)$.

9 $$\begin{aligned} f_2(T) = \dfrac{\text {EGC}_2(T)p_2^{EA}(T)}{2\text {GCD}_2(T)} \end{aligned}$$

The fecundity curves $f_1(T)$ and $f_2(T)$ are shown in Figure 2.

**Juvenile to adult development rates**

For the development rates we use the curves from Mordecai et al. (2017), which are based on the Brière curve for a trait *Y* that depends on temperature *T* (Jin et al. 2022; Briere et al. 1999):

$$\begin{aligned} Y(T) = c T (T-T_m)(T_M-T)^{1/2}. \end{aligned}$$

Here, *c* is a rate constant, and $T_m$ and $T_M$ are the thermal minimum and maximum values for the trait *Y*, respectively. The parameters *c*, $T_m$, and $T_M$ are estimated by fitting the equation for *Y* to thermal performance data for trait *Y*.

The development rates for both species increase with temperature. At extremely high temperatures, development rates may slow again, but there is a lack of data above 35$^\circ$C. Since there is no evidence of the development rates decreasing in this region, we extended the maximum values of the development rates:

$$\begin{aligned} d_{1}(T)=&{\left\{ \begin{array}{ll} \max (0.0000786T(T-11.35)(39.17-T)^{\frac{1}{2}},0), & T< 32.73\\ 0.1396, & T \ge 32.73\\ \end{array}\right. }\\ d_{2}(T)=&{\left\{ \begin{array}{ll} \max (0.0000638T(T-8.6)(39.66-T)^{\frac{1}{2}},0), & T < 32.73 \\ 0.1326, & {T \ge 32.73}. \end{array}\right. }\\ \end{aligned}$$

where the values of $d_i$ for $T\ge 32.73$ are calculated for $T=32.73$ in the given Brière functions.

**Adult mortality rates**

Curves were fit to both the $50 \%$ laboratory and field data for adult lifespan in Brady et al. (2013) (quadratic for *Ae. aegypti* and quartic for *Ae. albopictus*). The resulting equations are given below.

*Ae. aegypti* lifespan (field):

10 $$\begin{aligned} L_1^{\text {Field}}(T) = -0.007647T^2+0.3482T+1.844 \end{aligned}$$

*Ae. aegypti* lifespan (lab):

11 $$\begin{aligned} L_1^{\text {Lab}}(T) = -0.1194T^2+5.319T-18.16 \end{aligned}$$

*Ae. albopictus* lifespan (field):

12 $$\begin{aligned} L_2^{\text {Field}}(T) =0.0005404T^4+0.04539T^3-1.41T^2+19.45T-89.68 \end{aligned}$$

*Ae. albopictus* lifespan (lab):

13 $$\begin{aligned} L_2^{\text {Lab}}(T) = 0.0006021T^4-0.06474T^3+2.046T^2-19.26T+52 \end{aligned}$$

Adult mortality was then determined by taking the inverse of the average of equations (10) and (11) for *Ae. aegypti* and (12) and (13) for *Ae. albopictus*. A minimum lifespan of 1 day was assumed. The resulting adult mortality rates are thus

14 $$\begin{aligned} \mu _1(T) = \max {\left( \frac{2}{L_1^{\text {Lab}}(T) + L_1^{\text {Field}}(T)}, 1\right) } \end{aligned}$$

and

15 $$\begin{aligned} \mu _2(T) = \max {\left( \frac{2}{L_2^{\text {Lab}}(T) + L_2^{\text {Field}}(T)},1\right) } \end{aligned}$$

for *Ae. aegypti* and *Ae. albopictus*, respectively. Mortality rates for both species are shown in Figure 2.

### Disease Model Parameters

**Vector biting rates**

We define the vector biting rate for species *i* to be $a_i$. We consider both unscaled biting rates and biting rates scaled to incorporate different biting habits of *Ae. aegypti* and *Ae. albopictus*. We define the unscaled biting rates as in Mordecai et al. (2017) using Brière functions:

16 $$\begin{aligned} &  a_1^{\text {unscaled}}(T) = 0.000202T(T-13.35)(40.08-T)^{\frac{1}{2}} \end{aligned}$$

17 $$\begin{aligned} &  a_2^{\text {unscaled}}(T) = 0.000193T(T-10.25)(38.32-T)^{\frac{1}{2}}. \end{aligned}$$

The scaled biting rates are defined in terms of the unscaled biting rates as

18 $$\begin{aligned} a_1^{\text {scaled}}(T)= &  2 a_1^{\text {unscaled}}(T) \end{aligned}$$

19 $$\begin{aligned} a_2^{\text {scaled}}(T)= &  \frac{1}{2} a_2^{\text {unscaled}}(T). \end{aligned}$$

In the model, we set $a_i(T) = a_i^{\text {unscaled}}(T)$ to study the impacts of unscaled biting rates and $a_i(T) = a_i^{\text {scaled}}(T)$ to study impacts of scaled biting rates.

**Host to vector transmission probability per bite**

The Brière functions are from Mordecai et al. (2017) with $\rho _{v_1}$ modified above 31.04$^\circ$C to take on the value of the Brière function evaluated at $T=31.04$.

$$\begin{aligned} \rho _{v_1}(T)=&{\left\{ \begin{array}{ll} 0.000849T(T-17.05)(35.83-T)^{\frac{1}{2}}, & T < 31.04\\ 0.8069, & {T \ge 31.04} \end{array}\right. }\\ \rho _{v_2}(T)=&\ 0.000735T(T-15.84)(36.4-T)^{\frac{1}{2}} \end{aligned}$$

**Vector to host transmission probability per bite**

The Brière functions are from Mordecai et al. (2017) with $\rho _{h_1}$ modified above 31.22$^\circ$C to take on the value of the Brière function when $T=31.22$.

$$\begin{aligned} \rho _{h_1}(T) =&{\left\{ \begin{array}{ll} 0.000491T(T-12.22)(37.46-T)^{\frac{1}{2}}, & T < 31.22\\ 0.7275, & {T \ge 31.22}. \end{array}\right. }\\ \rho _{h_2}(T) =&\ 0.000439T(T-3.62)(36.82-T)^{\frac{1}{2}} \end{aligned}$$

**Vector extrinsic incubation rate**

For the incubation rate, we include the Brière functional forms from Mordecai et al. (2017).

$$\begin{aligned} \sigma _{v_1}(T)&= 0.0000665T(T-10.68)(45.9-T)^{\frac{1}{2}}\\ \sigma _{v_2}(T)&= 0.000109T(T-10.39)(43.05-T)^{\frac{1}{2}} \end{aligned}$$

**Temperature-independent parameters**

The remaining model parameters are taken to be temperature independent. For these parameters, we use the values from Huber et al. (2018).

Host intrinsic incubation period: $\sigma _h = 1/5.9$

Human recovery rate: $r_h = 1/5$

Birth/Death rate of humans: $\mu _h = 1/(70*365)$

Finally, we assume a human populations size of $N_h = 200$ for all simulations to obtain plausible vector-host ratios and $R_0$ values.
